# Children and adolescents with ASD treated with CBD-rich cannabis exhibit significant improvements particularly in social symptoms: an open label study

**DOI:** 10.1038/s41398-022-02104-8

**Published:** 2022-09-09

**Authors:** Micha Hacohen, Orit E. Stolar, Matitiahu Berkovitch, Odelia Elkana, Elkana Kohn, Ariela Hazan, Eli Heyman, Yael Sobol, Danel Waissengreen, Eynat Gal, Ilan Dinstein

**Affiliations:** 1 Azrieli National Centre for Autism and Neurodevelopment Research, Ben Gurion University, Beer Sheva, Israel; 2 Cognitive and Brain Sciences Department, Ben Gurion University, Beer Sheva, Israel; 3 The Academic College of Tel Aviv Yaffo, Tel Aviv, Israel; 4 ALUT Autism Center, Shamir Medical Center, Zerifin, Israel; 5 Clinical Pharmacology and Toxicology Unit, Shamir Medical Center, Zerifin, Israel; 6 Sackler School of Medicine, Tel-Aviv University, Tel Aviv, Israel; 7 Department of Pediatric Neurology, Shamir (Assaf Harofeh) Medical Center, Be’er Ya’akov, Israel; 8 Preschool Psychiatry Unit, Soroka Medical Center, Be’er Sheva, Israel; 9 Occupational Therapy Department, University of Haifa, Haifa, Israel

**Keywords:** Autism spectrum disorders, Clinical pharmacology, Human behaviour

## Abstract

In recent years there has been growing interest in the potential benefits of CBD-rich cannabis treatment for children with ASD. Several open label studies and one double-blind placebo-controlled study have reported that CBD-rich cannabis is safe and potentially effective in reducing disruptive behaviors and improving social communication. However, previous studies have mostly based their conclusions on parental reports without the use of standardized clinical assessments. Here, we conducted an open label study to examine the efficacy of 6 months of CBD-rich cannabis treatment in children and adolescents with ASD. Longitudinal changes in social communication abilities and restricted and repetitive behaviors (RRB) were quantified using parent report with the Social Responsiveness Scale and clinical assessment with the Autism Diagnostic Observation Schedule (ADOS). We also quantified changes in adaptive behaviors using the Vineland, and cognitive abilities using an age-appropriate Wechsler test. Eighty-two of the 110 recruited participants completed the 6-month treatment protocol. While some participants did not exhibit any improvement in symptoms, there were overall significant improvements in social communication abilities as quantified by the ADOS, SRS, and Vineland with larger improvements in participants who had more severe initial symptoms. Significant improvements in RRB were noted only with parent-reported SRS scores and there were no significant changes in cognitive scores. These findings suggest that treatment with CBD-rich cannabis can yield improvements, particularly in social communication abilities, which were visible even when using standardized clinical assessments. Additional double-blind placebo-controlled studies utilizing standardized assessments are highly warranted for substantiating these findings.

## Introduction

There are currently no approved medications for treating core autism spectrum disorders (ASD) symptoms, which include persistent deficits in social communication and restricted and repetitive patterns of behavior or interests [1]. However, children and adults with ASD are often prescribed medications to treat co-morbid symptoms, including hyperactivity, aggressiveness, irritability, anxiety, and sleep disturbances [2–4].

Endocannabinoids, including anandamide (AEA) and 2-arachidonoyl glycerol (2-AG), are lipid neuromodulators that regulate excitatory and inhibitory synaptic transmission through the activation of cannabinoid receptors and impact a variety of behavioral indices, including cognitive function, emotional regulation, social motivation, and reward processing [5]. Several recent reviews have suggested that treatment with cannabinoids may have the potential for improving core ASD symptoms as well as comorbid symptoms [6–8]. This suggestion is based on three lines of evidence. First, preclinical studies with mice models of ASD have reported that increasing the activity of AEA endocannabinoids in Fragile X and BTBR mice [9] and increasing the activity of 2-AG in SHANK3B mice [10] significantly improved their social impairments. Similarly, increasing AEA levels in female prairie voles increased their social interactions [11] and administration of phyto-cannabinoids to SHANK3 mice significantly reduced anxiety and repetitive grooming symptoms [12]. The second line of evidence comes from studies reporting that at least some children with ASD exhibit significantly lower plasma levels of endocannabinoids including AEA [13], *N*-palmitoylethanolamine, and *N*-oleoylethanolamine [14].

The third, and perhaps most convincing line of evidence comes from clinical studies with ASD participants. In the last two decades parents of children with ASD have reported anecdotal success in autonomously treating their children with medicinal cannabis despite a lack of clinical guidelines on the topic [15]. Following the legalization of cannabis for medicinal use in many western countries and its approval for compassionate use in ASD, several open label studies have reported that children with ASD respond well to treatment with CBD-rich cannabis and that this treatment is both safe and effective [16–19]. More specifically, several studies using parent questionnaires have reported improvements in social communication [16–21], while others have reported reductions in disruptive behaviors, including self-injury, tantrums, restlessness, and agitation [17, 21].

To date, only one double-blind placebo-controlled study has tested the effectiveness of CBD-rich cannabis for treating ASD. The study examined 150 children and adolescents with ASD, 5–21 years old, over a treatment period of three months. The results revealed that the treatment was safe and effective even in improving core ASD symptoms as reported by parent questionnaires and clinical assessment using the Clinical Global Impression scale [16].

Note that the choice to treat individuals with ASD using CBD-rich cannabis, as performed in the studies described above, was motivated by concerns that THC-rich cannabis would induce psychosis [22, 23]. Furthermore, CBD-rich cannabis was proven safe and effective in treating epileptic seizures in children [24] and was reported to improve symptoms in children with ASD and epilepsy [19, 21].

The aim of the current study was to expand existing knowledge by performing an open label study of treatment efficacy with CBD-rich cannabis. Unlike previous studies that have relied almost entirely on parent report, here we performed standardized clinical behavioral assessments alongside parental reports, before and after 6 months of treatment. This enabled us to quantify the effects of treatment separately for social communication and restricted and repetitive behaviors, adaptive behaviors, and cognitive abilities, while examining the correspondence between clinical assessments and parental reports.

## Methods

### Participants and recruitment procedure

We analyzed data from 82 participants who completed the study (see below). A total of 110 participants (65 male, mean age: 9.2 years old, range: 5–25 years old) were recruited to this prospective study that was carried out at Shamir Medical Center in Israel. Families were recruited through advertisements in the community. Upon recruitment, a pediatric neurologist, who specializes in ASD, interviewed the parents about the child’s medical history. Participants were included in the study if they fulfilled the DSM-5 criteria for ASD and reported disruptive behavioral problems over the duration of the preceding 6 months. Criteria for exclusion included: any use of cannabis prior to the study, the previous diagnosis of a genetic disorder, active epilepsy, a metabolic disease, an immunological disease, current use of opiates, being pregnant or breastfeeding, diagnosis of the participant or a first-degree family member with psychosis, schizophrenia, schizoaffective disorder, or substance abuse. All participants also completed a video EEG assessment to rule out epilepsy.

Participants who were taking routine medications were instructed to continue without making any changes during the study period. Out of 110 participants, 42 participants were regularly taking at least one medication (Table 1). Both parents, or a legal guardian, of each participant signed an informed consent form. The study was approved by both institutional and national ethics Committees, and was registered with a clinical trial number in the Israeli ministry of health (MOH) as trail number: MOH_01_02_2019_004876.

**Table 1:** Chronic medications taken by participants during the study.

Reason for treatment	Medication generic name	*N*
Sleep problems	Melatonin	14
Irritability, aggressive behavior	Aripiprazole	9
	Risperidone	7
	Coltiapine	2
	Periciazine	2
	Levomepromazine	1
	Periciazine	1
	Quetiapine	1
Hyperactivity and attention deficit disorder	Methylphenidate	5
	Lisdexamfetamine	4
	Atomoxetine	2
	Dextroamphetamine	1
	Guanfacine	1
Anxiety	Fluoxetine	4
	Clonazepam	2
Other	Dimethindene	1

### Behavioral assessments

Participants completed the following behavioral assessments before starting the treatment and again six months later (Table 2). In total, 53 participants completed all behavioral assessments and 29 completed partial assessments. All participants who dropped out of the study (Table 3) did not participate in assessments at the end of the study due to poor motivation and despite invitations to participate. The assessments examined in this study included:

**Table 2:** Characteristics of participants who completed the study at treatment onset.

	Mean (standard deviation)
Age	9.3 (0.5)
ADOS CSS (*n* = 75)	8.44 (0.2)
SRS total score (*n* = 61)	111.62 (3.17)
Vineland total score (*n* = 76)	56.51 (1.9)
Cognitive GAI (*n* = 76)	62.03 (3.16)

Mean and standard deviation in parenthesis.

**Table 3:** Characteristics of participants who dropped out of the study.

	Age	Sex	Final CBD dosage (mg/kg)	Dropout reason
Lack of cooperation (*n* = 8)	11.1 (1.2)	7M/1F	1.55 (0.26)	3 refused to consume cannabis oil 2 stopped treatment for >7 days 2 refused to give blood samples 1 family did not fill questionnaires
Side effects (*n* = 12)	9.9 (2)	9M/3F	1.58 (0.323)	5 increased aggression 3 increased anxiety 1 weight gain 1 abdominal pain 1 increased Hyperactivity 1 decrease in communication
No improvement (*n* = 8)	8.4 (1.1)	5M/3F	2.2 (0.54)	
Total (*n* = 28)	9.7 (1)	21M/7F	1.76 (0.22)	

Mean and standard deviation in parenthesis.

#### Autism diagnostic observation schedule, 2nd edition (ADOS-2)

Of the 82 participants who completed the study, 75 completed the ADOS-2 [25]. We used the calibrated severity scores (CSS), which are a transformation of the total ADOS-2 raw scores into a scale of 0–10 that represents the child’s ASD symptom severity regardless of the administered ADOS module (i.e., regardless of the child’s age and language abilities). CSS scores are available for the total ADOS-2 and for the Social Affect (SA) and Restricted and Repetitive Behavior (RRB) domains separately [26]. The ADOS-2 assessment was performed by a trained and licensed speech therapist with research reliability.

#### Cognitive assessments

Seventy-six participants completed five subtests of an age-appropriate Wechsler Intelligence Scale; WPPSI [27], WISC [28] or WAIS [29]. Cognitive assessments of children above the age of 6 years old included the Block design and Matrix subtests from the Perceptual Organization Index (POI), the Vocabulary and Similarities subtests from the Verbal Comprehension Index (VCI) and the Digit symbol-coding subtest from the Processing Speed Index (PSI). Children below the age of 6 years old performed the Information subtest instead of the Similarities subtest. We assessed changes in the standardized scores of each sub-test, which estimate cognitive abilities relative to typical age norms that have a population mean of 10 and a standard deviation of 3.

#### Vineland adaptive behaviors scale, 3rd edition (Vineland-3)

Seventy-six participants completed the survey form Vineland-3 [30], which was administered as a parent interview by a trained research assistant. The Vineland scale assesses adaptive functions in four domains: Communication, Daily Living Skills, Socialization, and Motor Skills. Raw scores from each domain are transformed to age equivalent standardized scores with a population mean of 100 and a standard deviation of 15. Standardized scores of the communication, daily living skills, and socialization domains are summed to create the overall Adaptive Behavior Composite Score (ABC).

#### Social responsiveness scale, 2nd edition (SRS-2)

Sixty-one participants completed the SRS-2 [31]. The SRS is a 65-item questionnaire completed by parents. The questions focus on the child’s behavior over the last 6 months and provide information about social skills including social awareness, social cognition, social communication, social motivation, and restricted and repetitive behaviors. The cutoff for clinical ASD symptoms is typically a score above 60, with scores in the range of 60–75 indicating mild to moderate ASD symptoms, and scores over 75 indicating severe symptoms. All four social sub-scales can be summed and standardized to a single score representing social abilities, separately from the scores of the RRB scale.

### Drop out

Twenty-eight of the 110 participants who started treatment, did not complete the study. Eight were excluded due to lack of corporation of the child or the family with one or more of the study procedures (e.g., inability to consume cannabis regularly), 12 stopped treatment due to adverse side effects, and 8 stopped because of lack of improvement (Table 3).

### Cannabis treatment protocol

Parents received a supply of medicinal cannabis whole-plant extract infused in medium-chain triglyceride (MCT) oil with a CBD:THC ratio of 20:1 (Nitzan Spectrum®, Seach Medical Group, Israel) for a period of six months. The exact same product was used throughout the treatment period. Parents were instructed to start with one drop daily (each drop contains: 0.3 mg THC and 5.7 mg CBD) and increase the dosage gradually until they perceived improvements in their child’s behavior such as decreased irritability, aggressiveness, hyperactivity, and/or sleep disturbances. The amount and timing of doses during each day was tailored to individual needs of the child (e.g., higher dose at night if needed for sleep support). Parents completed a bi-weekly phone interview where they reported compliance, behavior, symptoms, and side effects. The final dose did not exceed 10 mg/kg/day (or total of 400 mg/day) of CBD and 0.5 mg/kg/day (or total of 20 mg/day) of THC.

### Statistics

Statistical analyses were performed using JASP (Version 0.14.1.0) and R studio (Version 1.1.466). By recruiting >100 subjects our study had a power of >0.9 to identify a moderate treatment effect size of *d* = 0.5. Since data from most of the measures was not normally distributed (see table in supplement 1), a related-samples randomization test was performed to determine whether scores at the end of the treatment period differed significantly from pre-treatment scores [32]. Actual pre/post differences were compared to a null distribution of 10,000 random pre/post treatment differences. This distribution was generated by computing the pre/post treatment difference for each subject and then randomly shuffling its sign (positive or negative) before computing the mean across the group. This manipulation retained the magnitude of change for each subject while randomizing its direction. To achieve statistical significance the actual mean change of the group had to exceed the 95th percentile of this distribution (i.e., equivalent to a *p* value of 0.05). Note that this is a more conservative statistical test than a paired *t*-test, which assumes that pre/post treatment differences are normally distributed. Multiple regression analyses were used to determine the influence of several covariates on pre/post treatment differences.

### Missing data

There were missing items in some of the collected SRS questionnaires. Questionnaires with more than 25% of missing data (i.e., more than 16 of the 65 items) were excluded from analysis. In the remainder of cases, missing data were completed using the multivariate imputation by chained equation (MICE) package as implemented in the R software [33]. In short, this technique creates imputations (replacement values) based on the mean values of available responses for each item and a linear regression analysis that estimates the relationship of each item with all others.

## Results

**Fig. 1:**
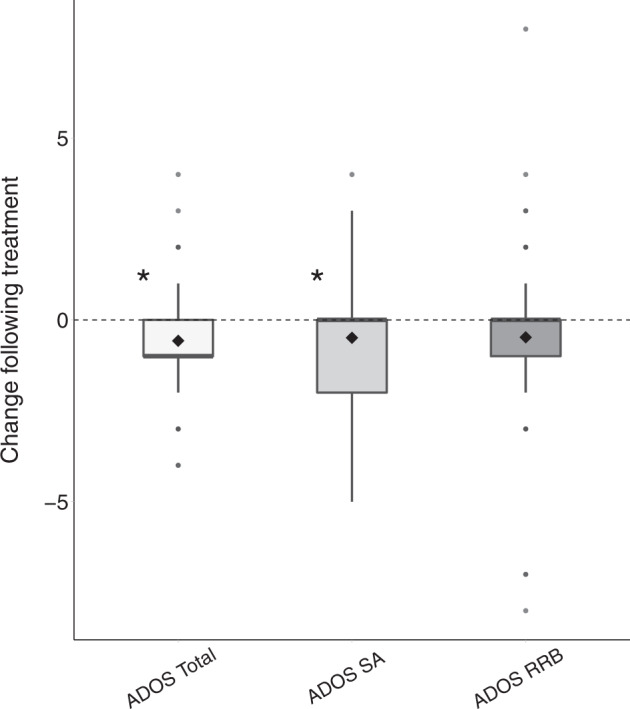
Change in ADOS CSS following 6-month cannabis treatment. Box plot diagrams presenting distribution of changes in the total ADOS CSS (left) ADOS CSS SA (middle) and ADOS CSS RRB (right). Asterisk: significant change (*p* < 0.05, randomization test). Black diamond: mean. Bold line: median.

Of the 82 participants who completed the study, 75 completed ADOS assessments before and after 6 months of treatment. There was a significant improvement in the ADOS total calibrated severity scores (CSS) of these participants (*M* = −0.56, SD = .17, Sum(*x*) = 42, *p* = 0.003, Fig. 1). Separating the ADOS CSS into social affect (SA) and restricted and repetitive behavior (RRB) components revealed that changes were driven by large improvements in ADOS SA CSS (*M* = −0.49, SD = .18, Sum(*x*) = 37, *p* = 0.001) and weak improvements in ADOS RRB CSS that were not significant (*M* = −0.47, SD = 0.26, Sum(*x*) = 42, *p* = 0.08).

**Fig. 2:**
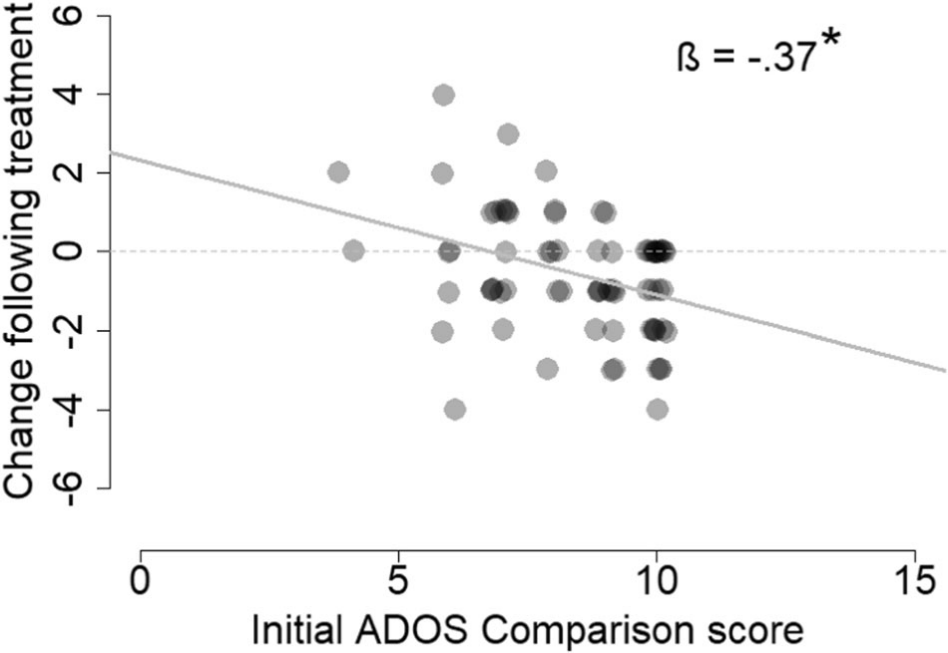
Relationship between initial ADOS scores and change pre/post treatment. Scatter plot demonstrating the relationship between initial ADOS CSS and change in ADOS CSS pre/post treatment. Asterisk: significant relationship (*p* < 0.05, linear regression). Gray line: regression line.

Next, we performed a multiple regression analysis to determine whether pre/post treatment changes in ADOS CSS were associated with the age, final dosage, and initial ADOS CSS of the participants. The results revealed that only initial ADOS CSS were significantly associated with change in the total ADOS CSS (β = −0.37, *p* = 0.002; Fig. 2) such that participants with higher initial ADOS CSS (i.e., more severe initial symptoms) exhibited larger improvements. Age (*β* = 0.03, *p* = 0.78) and final dosage (*β* = 0.03, *p* = 0.8) were not significantly associated with changes in the ADOS CSS. Equivalent findings were also apparent for the ADOS SA CSS and RRB CSS (see Supplementary material for detailed regression analysis of all measures).

**Fig. 3:**
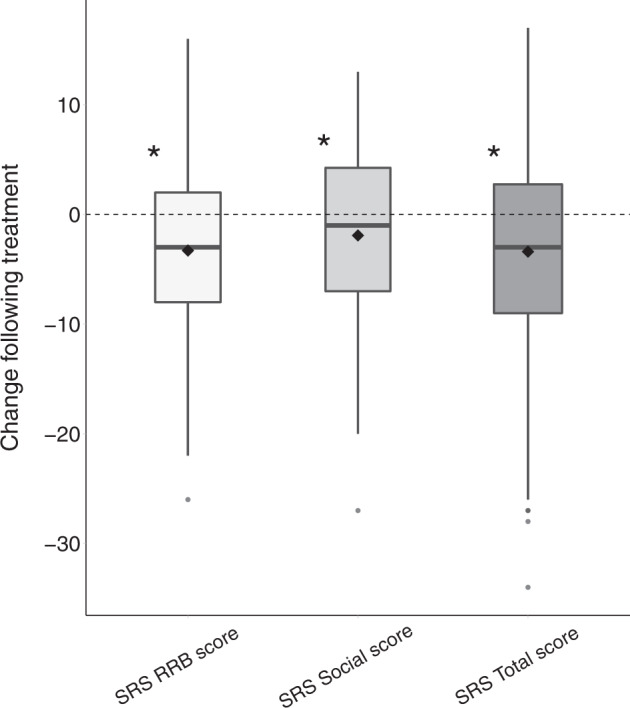
Change in SRS scores, before and after treatment. SRS restricted and repetitive behaviors (RRB) scale, SRS social scale, SRS total score. Asterisk: Significant change (*p* < 0.05, randomization test). Black diamond: mean. Bold line: median.

Of the 82 subjects who completed the study, parents of 61 completed the SRS-2 questionnaire before and after treatment. Parents reported a significant improvement in core ASD symptoms of children who completed treatment (*M* = −3.29, SD = 1.13, Sum*(x)* = 201, *p* = 0.043, Fig. 3). This was true for both the social sub-scale scores (*M* = −2.51, SD = 1.19, Sum*(x)* = 153, *p* = 0.038, Fig. 3) and the RRB sub-scale scores (*M* = −2.88, SD = 1.14, Sum(*x*) = 176, *p* = 0.014). A multiple regression analysis revealed that pre/post changes in SRS scores were significantly associated with the initial social (*β* = −0.49, *p* < .001) and RRB (*β* = −0.39, *p* = 0.003) scores both not with age (*β* = 0.04, *p* = 0.72) or final dosage (*β* = 0.08, *p* = 0.53). Hence, higher initial SRS scores predicted larger improvements following treatment.

**Fig. 4:**
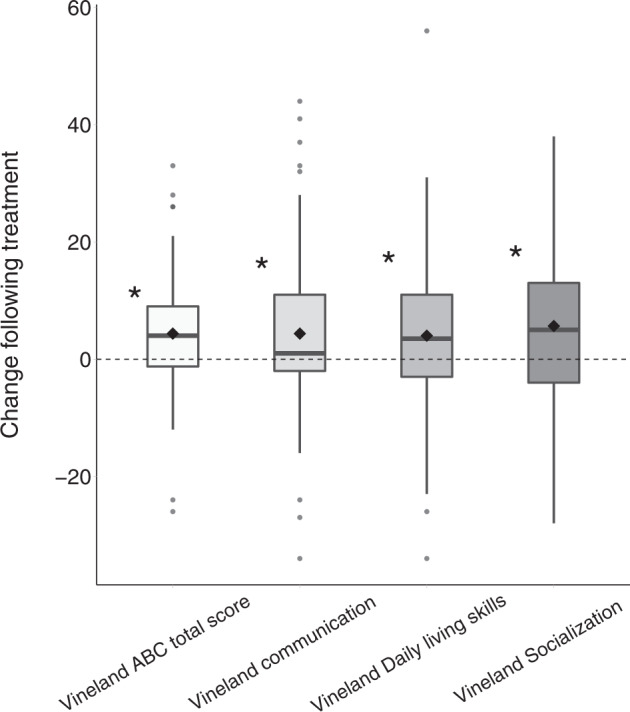
Change in Vineland scores pre/post treatment. Vineland total score, communication sub-scale, socialization sub-scale, daily living skills sub-scale. Asterisk: significant change (*p* < 0.05, randomization test). Black diamond: mean. Bold line: median.

Of the 82 subjects who completed the study, 76 completed the Vineland questionnaire before and after 6 months of treatment. There was a significant improvement in total Vineland scores in children who completed treatment (*M* = 4.37, SD = 1.18, Sum(*x*) = 332, *p* < 0.001, Fig. 4). Improvements were apparent in the communication (*M* = 4.37, SD = 1.61, Sum(*x*) = 332, *p* = 0.008), daily living (*M* = 4, SD = 1.47, Sum(*x*) = 305, *p* = 0.007), and socialization (*M* = 5.66, SD = 1.5, Sum(*x)* = 430, *p* < 0.001) sub-scales. A multiple regression analysis revealed that pre/post treatment changes in the socialization sub-scale were significantly associated with initial socialization sub-scale scores (*β* = −0.41, *p* < 0.001), but not with age (*β* = 0.06, *p* = 0.26) or dosage level (*β* = −0.01, *p* = 0.65). Participants with lower initial socialization scores exhibited larger improvements. Changes in other sub-scales of the Vineland questionnaire were not associated with any of the covariates.

**Fig. 5:**
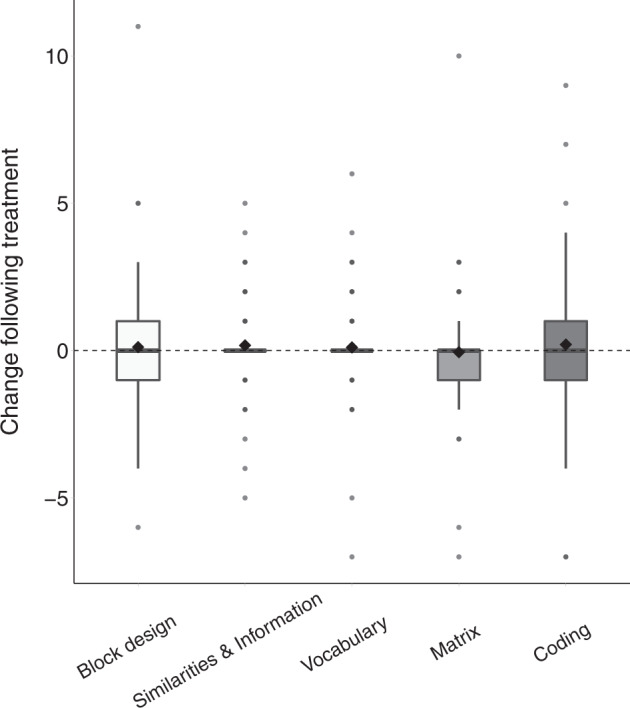
Change in cognitive sub-test scores pre/post treatment. Block design perceptual test, matrix perceptual test, vocabulary verbal test, similarities & information verbal test, coding processing speed test. Asterisk: significant change (*p* < 0.05, randomization test). Black diamond: mean. Bold line: median.

Of the 82 participants who completed the study, 76 completed cognitive assessments before and after 6 months of treatment. Equivalent analyses did not reveal any significant impact of treatment on any of the cognitive subtests: Block design perceptual (*M* = .13, SD = .25, Sum(*x*) = 10, *p* = 0.65, Fig. 5), Matrix perceptual (*M* = −0.14, SD = .19, Sum(*x*)=11, *p* = 0.5), Vocabulary verbal (*M* = 0.29, SD = .28, Sum(*x*) = 22, *p* = 0.37), Similarities & Information verbal (*M* = 0.04, SD = 0.22, Sum(*x*) = 3, *p* = 0.91), or Coding processing speed (*M* = 0.22, SD = 0.18, Sum(*x*) = 11, *p* = 0.24).

**Fig. 6:**
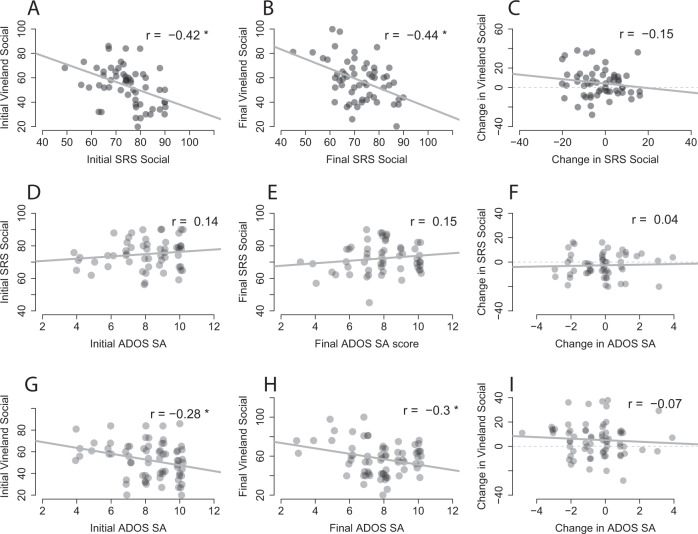
Relationship across different measures of social communication skills. Scatter plots demonstrate the relationship between: **A** Initial Vineland socialization and SRS social scores; **B** Final Vineland socialization and SRS social scores; **C** Pre/post change in Vineland socialization and SRS social scores. **D** Initial SRS social and ADOS-SA CSS scores; **E** Final SRS social and ADOS-SA CSS scores; **F** Pre/post change in SRS social and ADOS-SA CSS scores. **G** Initial ADOS-SA CSS and Vineland socialization scores; **H** Final ADOS-SA CSS and Vineland socialization scores; **I** Pre/post change in ADOS-SA CSS and Vineland socialization scores. Asterisk: significant correlation (*p* < 0.05). Gray line: linear least-squares fit.

Next, we examined the relationships between the SRS, Vineland, and ADOS, which measure similar ASD symptom domains in different manners (Fig. 6). There were significant correlations between scores from the socialization sub-scale of the Vineland and the SRS social scale both before (*r*(55) = −0.42, *p* = 0.001; Fig. 6) and after treatment (*r*(55) = −0.44, *p* < 0.001). However, there were no significant correlations in pre-post treatment changes across the two scales (*r*(55) = −0.015, *p* = 0.27) indicating low reliability in parental reported changes across the two measures.

Similarly, there were significant correlations between the ADOS-2 SA CSS and the Vineland socialization sub-scale scores before (*r*(67) = −0.28, *p* = 0.02) and after (*r*(67) = −0.3, *p* = 0.01) treatment, but there was no correlation in pre-post treatment changes across the two scales (*r*(67)=−0.07, *p* = 0.56). There were no significant correlations between the ADOS-2 SA CSS and SRS social scores before (*r*(54) = −0.14, *p* = 0.3) or after (*r*(54) = −0.15, *p* = 0.28) treatment and no correlation in pre-post treatment changes across the two measures (*r*(54) = 0.04, *p* = 0.77). This demonstrates low reliability between parental and clinical reports of changes in the severity of social symptoms following treatment.

## Discussion

Our results revealed significant improvements in overall ADOS-2, SRS, and Vineland scores of the ASD participants who completed the 6-month treatment protocol with CBD-rich cannabis. Overall changes were mostly driven by improvements in social communication skills that were apparent in ADOS-2 SA CSS (Fig. 1), SRS social scores (Fig. 3), and Vineland communication and socialization scores (Fig. 4). Significant improvements in RRB symptoms were apparent only in parent reports with the SRS (Fig. 3) and not in clinical reports with the ADOS-2 RRB CSS (Fig. 1). Regression analyses revealed that participants with more severe initial symptoms as measured by ADOS-2, Vineland, or SRS scores, exhibited larger improvements following intervention (Fig. 2) regardless of their age or final cannabis dosage. Treatment did not have a significant impact on any of the examined cognitive sub-tests (Fig. 5), indicating that cannabis treatment did not have a positive or negative impact on cognitive abilities.

Despite these positive results it is important to note three caveats. First, these results are based on data from 82 of 110 participants who started the study. Of the 28 participants who did not complete the study for various reasons (Table 3), 12 participants (i.e., 11% of the initial sample) stopped treatment due to adverse side effects. Since these participants did not complete behavioral assessments at the end of the treatment period, we do not know the potential impact of these missing data on our results. Second, the median change in ADOS-2 SA scores following treatment was zero (Fig. 1). This indicates that roughly half of the participants (51%) who completed treatment did not exhibit improvements in core ASD symptoms as measured by the ADOS-2 SA CSS. Hence, significant group improvements in ADOS-2 SA scores were driven by large improvements that were reported by less than half of the participants who completed the study. Third, there was notable inconsistency across measures of change in social communication skills following treatment, with no significant correlations across ADOS-2 SA, Vineland communication and socialization, and SRS social scores (Fig. 6). This suggests that improvements were noted for different participants when using different measures, indicating that parents and clinicians did not report consistent changes in the social behavior of individual participants.

Nevertheless, these findings suggest that treatment with CBD-rich medicinal cannabis can lead to significant improvements in social communication skills of some ASD individuals, particularly those with more severe initial symptoms. Moreover, these improvements were large enough to be visible even when using coarse standardized clinical assessments such as the ADOS-2.

### Effect of cannabis treatment on core ASD symptoms

The results of our study are in line with several previous studies demonstrating the potential efficacy of CBD-rich medicinal cannabis for treatment of ASD. These include studies reporting that cannabis treatment was effective in improving social communication skills as reported by parents [16, 18–21]. Our results extend these findings by demonstrating that improvement in social communication skills was apparent not only in parent reported SRS scores, but also in clinical scores using the relatively coarse ADOS-2 SA CSS scale that has a range of 0–10. In contrast, improvements in RRB symptoms were apparent only in the parent reported SRS scores, but not in ADOS-2 RRB CSS. Hence, of the two core ASD symptoms, treatment with CBD-rich cannabis seems to have a more consistently reported positive effect on social symptoms and particularly in cases with more severe initial social symptoms.

While previous studies focused entirely on group effects (i.e., how treatment affected the group on average), we also examined the reliability of individual treatment effects across multiple measures of social communication. This revealed that parent report and clinical observation measures were poorly correlated (Fig. 6), indicating that different magnitudes of change were reported for different participants when using different measures/tools. This raises concerns regarding the reliability of reported social communication treatment effects and highlights the need for substantiating and implementing more objective outcome measures that can directly assess the severity of core ASD symptoms (e.g., using analysis of speech recordings [34] or eye tracking protocols [35].

### Effect of cannabis treatment on additional behavioral domains

We also examined changes in adaptive behaviors and cognitive abilities, two behavioral domains that have not been examined to date in Cannabis treatment studies. The results revealed a significant improvement in adaptive behaviors as measured by the Vineland. This overall improvement was primarily driven by improvements in social, communication, and daily living sub-scale scores that were all significant. In contrast, cognitive assessments did not reveal any changes following treatment. These results demonstrate that CBD-rich cannabis treatment at least does not seem to have detrimental effects on cognitive function.

### Potential mechanisms of action

The cannabis plant includes more than 100 cannabinoids (phytocannabinoids), which vary in their relative concentrations across strains. While cannabis has been used for medicinal and recreational purposes for thousands of years, the individual and entourage effects of different cannabinoids on the human body are poorly understood. Most research to date has focused on the effects of two cannabinoids: Δ9-tetrahydrocannabinol (THC) and cannabidiol (CBD). THC is a partial agonist of the cannabinoid receptor (CB1) in the brain and induces the psychoactive effects of cannabis [36]. CBD is an allosteric modulator of the CB1 receptor and has an analgesic effect that counteracts and complements the effects of THC [37].

Initial studies with recreational cannabis smokers revealed that within controlled settings, smokers were more interactive, communicative, comfortable, and open toward one another [38] and exhibited enhanced social cooperation and reduced hostility [39], compared with nonsmokers. While these studies suggest that cannabis may improve some aspects of social communication, perhaps through the activation of Oxytocin related pathways [9], the specific strains used in these studies and their cannabinoid compositions were not examined or controlled. To date, treatment of individuals with ASD has mostly been carried out with CBD-rich cannabis, because of the analgesic quality and low risk of generating psychotic events, in contrast to THC-rich cannabis [40]. However, comparison of the behavioral impact of different strains and/or specific cannabinoids has not been performed systematically and considerable research is required for revealing their underlying mechanisms of action [41]. Such research will hopefully enable future identification of the cannabinoid, or cannabinoid group, that most benefit individuals with ASD.

## Limitations

The current study had several limitations that should be acknowledged. First and foremost, this was an open label study, which is likely to create a positive bias in parent and clinician reports given known placebo effects [42, 43]. Indeed, a recent double-blind placebo-control study, testing cannabis treatment for children with ASD, reported that 21% of the participants showed improvement in clinical assessment after receiving placebo treatment [16]. Second, out of the total 110 participants who started the treatment, 28 individuals did not complete the treatment protocol for different reasons (Table 3). It is possible that if these participants had completed the study, the reported improvement would have been milder or would not have achieved statistical significance. In addition, due to COVID-19 restrictions, some parents failed to complete follow-up questionnaires and clinical assessments in time, which resulted in missing data in some of the measures. As a result, out of the 82 participants who completed the treatment protocol, only 53 completed all four behavioral assessments. Since different participants completed different sets of assessments, the ability to observe and compare trends of behavioral changes in the entire study sample was reduced. Third, participants were recruited through advertisements in Hebrew and participation in the study required travel to a medical center. This may have created a bias in our sample towards higher socio-economic Hebrew speaking participants. Last, the dose of cannabis was adjusted individually such that each participant received a different dosage schedule throughout the study. While this approach allowed maximum flexibility to the needs of each participant and their family, it limited the ability to accurately monitor the influence of different dosages and dosage schedules and associate them with efficacy. More stringent double-blind placebo-controlled studies with comparable dosage schedules are, therefore, highly warranted for determining efficacy using the standardized behavioral assessments presented in the current study.

## Conclusion

Accumulating evidence, mostly from open-label uncontrolled studies suggest that CBD-rich cannabis may yield benefits for some individuals with ASD. In this study we demonstrate that this benefit includes improvement in social communication abilities, particularly for participants with high initial severity of core ASD symptoms. Moreover, this is the first study to examine the efficacy of cannabis treatment using both standardized clinical assessments (i.e., ADOS), parent interviews (i.e., Vineland) and questionnaires (i.e., SRS). Despite differences in individual scores reported by parents and clinicians (Fig. 6), the convergence of evidence regarding overall improvements following treatment strengthens the conclusions. These positive findings motivate further double-blind placebo-controlled studies for determining the efficacy of treatment with specific cannabis strains and/or synthetic cannabinoids.

## Supplementary information

Cannabis treatment paper Supplementary Materials
