# Supplementary material for: Children and adolescents with ASD treated with CBD-rich cannabis exhibit significant improvements particularly in social symptoms: an open label study
Source: Transl Psychiatry. 2022 Sep 9;12:375. doi: 10.1038/s41398-022-02104-8 (PMC9461457; doi:10.1038/s41398-022-02104-8)
Supplement: Supplementary file 1 — Cannabis treatment paper Supplementary Materials [file 41398_2022_2104_MOESM1_ESM.docx]

**Children and adolescents with ASD treated with CBD-rich cannabis exhibit significant improvements particularly in social symptoms: an open label study**

Micha Hacohen^1,2,3^*, Orit E. Stolar^4^*, Matitiahu Berkovitch^5,6^, Odelia Elkana^3^, Elkana Kohn^5,6^, Ariela Hazan^5^, Eli Heyman^5^, Yael Sobol^8^, Danel Waissengreen^1^, Eynat Gal^9^, and Ilan Dinstein^1,2^

1. Azrieli National Centre for Autism and Neurodevelopment Research, Ben Gurion University, Beer Sheva
2. Cognitive and Brain Sciences Department, Ben Gurion University
3. The Academic College of Tel Aviv Yaffo, Tel Aviv
4. ALUT Autism Center, Shamir Medical Center, Zerifin
5. Department of Pediatric Neurology, Shamir (Assaf Harofeh) Medical Center
6. Clinical Pharmacology and Toxicology Unit, Shamir Medical Center, Zerifin
7. Sackler School of Medicine, Tel-Aviv University, Tel Aviv
8. Preschool Psychiatry Unit, Soroka Medical Center
9. Occupational Therapy Department, University of Haifa, Haifa

*** Equal contribution as first authors**

| **Supplementary table 1:**  Regression analysis predicting change in ADOS total CSS using age, final dosage, and initial ADOS CSS of the participants as predictors. | | | | | | | | | | | | | |  |
| --- | --- | --- | --- | --- | --- | --- | --- | --- | --- | --- | --- | --- | --- | --- |
| **Model** | |  | | **B** | **SE** | | **β** | | ***t*** | | ***p*** | | |  |
| H₀ |  | (Intercept) |  | -0.57 |  | 0.18 |  |  |  | -3.22 | |  | 0.002 |  |
| H₁ |  | (Intercept) |  | 2.11 |  | 1.04 |  |  |  | 2.03 | |  | 0.046 |  |
|  |  | Dosage |  | 0.06 |  | 0.15 |  | 0.045 |  | 0.37 | |  | 0.72 |  |
|  |  | Age |  | 0.01 |  | 0.04 |  | 0.043 |  | 0.35 | |  | 0.73 |  |
|  |  | Initial ADOS total CSS |  | -0.35 |  | 0.11 |  | -0.37 |  | -3.2 | |  | 0.002 |  |

| **Supplementary table 2:**  Regression analysis predicting change in ADOS social affect (SA) CSS using age, final dosage, and initial ADOS SA CSS of the participants as predictors. | | | | | | | | | | | | | |  |
| --- | --- | --- | --- | --- | --- | --- | --- | --- | --- | --- | --- | --- | --- | --- |
| **Model** | |  | | **B** | **SE** | | **β** | | ***t*** | | ***p*** | | |  |
| H₀ |  | (Intercept) |  | -0.5 |  | 0.18 |  |  |  | -2.8 | |  | 0.007 |  |
| H₁ |  | (Intercept) |  | 2.69 |  | 0.94 |  |  |  | 2.85 | |  | 0.006 |  |
|  |  | Dosage |  | -0.26 |  | 0.15 |  | -0.208 |  | -1.7 | |  | 0.093 |  |
|  |  | Age |  | -0.03 |  | 0.04 |  | -0.074 |  | -0.6 | |  | 0.55 |  |
|  |  | Initial ADOS SA CSS |  | -0.27 |  | 0.1 |  | -0.318 |  | -2.8 | |  | 0.006 |  |

| **Supplementary table 3:**  Regression analysis predicting change in ADOS restricted repetitive behavior (RRB) CSS using age, final dosage, and initial ADOS RRB CSS of the participants as predictors. | | | | | | | | | | | | | | |  |
| --- | --- | --- | --- | --- | --- | --- | --- | --- | --- | --- | --- | --- | --- | --- | --- |
| **Model** | |  | | **B** | **SE** | | **β** | | ***t*** | | ***p*** | | | |  |
| H₀ |  | (Intercept) |  | -0.47 |  | 0.26 |  |  |  | -1.8 | |  | 0.08 |  | |
| H₁ |  | (Intercept) |  | 4.13 |  | 1.46 |  |  |  | 2.83 | |  | 0.01 |  | |
|  |  | Dosage |  | 0.49 |  | 0.2 |  | 0.27 |  | 2.41 | |  | 0.02 |  | |
|  |  | Age |  | 0.04 |  | 0.06 |  | 0.08 |  | 0.72 | |  | 0.47 |  | |
|  |  | Initial ADOS RRB CSS |  | -0.74 |  | 0.14 |  | -0.54 |  | -5.31 | |  | < .001 |  | |

| **Supplementary table 4:**  Regression analysis predicting change in SRS total scaled score using age, final dosage, and initial SRS total scaled score of the participants as predictors. | | | | | | | | | | | | | |  |
| --- | --- | --- | --- | --- | --- | --- | --- | --- | --- | --- | --- | --- | --- | --- |
| **Model** | |  | | **B** | **SE** | | **β** | | ***t*** | | ***p*** | | |  |
| H₀ |  | (Intercept) |  | -3.29 |  | 1.13 |  |  |  | -2.97 | |  | 0.005 |  |
| H₁ |  | (Intercept) |  | 32.24 |  | 9.66 |  |  |  | 3.34 | |  | 0.001 |  |
|  |  | Dosage |  | 0.65 |  | 1.03 |  | 0.08 |  | 0.64 | |  | 0.53 |  |
|  |  | Age |  | 0.1 |  | 0.28 |  | 0.04 |  | 0.36 | |  | 0.72 |  |
|  |  | Initial SRS total score |  | -0.49 |  | 0.13 |  | -0.48 |  | -3.88 | |  | < .001 |  |

| **Supplementary table 5:**  Regression analysis predicting change in SRS social scaled score using age, final dosage, and initial SRS social scaled score of the participants as predictors. | | | | | | | | | | | | | |  |
| --- | --- | --- | --- | --- | --- | --- | --- | --- | --- | --- | --- | --- | --- | --- |
| **Model** | |  | | **B** | **SE** | | **β** | | ***t*** | | ***p*** | | |  |
| H₀ |  | (Intercept) |  | -2.52 |  | 1.21 |  |  |  | -2.08 | |  | 0.04 |  |
| H₁ |  | (Intercept) |  | 37.62 |  | 8.97 |  |  |  | 4.19 | |  | < .001 |  |
|  |  | Dosage |  | 1.13 |  | 0.99 |  | 0.14 |  | 1.14 | |  | 0.26 |  |
|  |  | Age |  | -0.02 |  | 0.29 |  | -0.01 |  | -0.08 | |  | 0.94 |  |
|  |  | Initial SRS social score |  | -0.57 |  | 0.12 |  | -0.56 |  | -4.87 | |  | < .001 |  |

| **Supplementary table 6:**  Regression analysis predicting change in SRS RRB scaled score using age, final dosage, and initial SRS RRB scaled score of the participants as predictors. | | | | | | | | | | | | | |  |
| --- | --- | --- | --- | --- | --- | --- | --- | --- | --- | --- | --- | --- | --- | --- |
| **Model** | |  | | **B** | **SE** | | **β** | | ***t*** | | ***p*** | | |  |
| H₀ |  | (Intercept) |  | -2.8 |  | 1.16 |  |  |  | -2.42 | |  | 0.02 |  |
| H₁ |  | (Intercept) |  | 25.58 |  | 11.6 |  |  |  | 2.2 | |  | 0.03 |  |
|  |  | Dosage |  | -0.05 |  | 1.07 |  | -0.01 |  | -0.05 | |  | 0.96 |  |
|  |  | Age |  | 0.23 |  | 0.3 |  | 0.1 |  | 0.77 | |  | 0.44 |  |
|  |  | Initial SRS RRB score |  | -0.38 |  | 0.14 |  | -0.34 |  | -2.66 | |  | 0.01 |  |

| **Supplementary table 7:**  Regression analysis predicting change in Vineland communication scaled score using age, final dosage, and initial Vineland communication score of the participants as predictors. | | | | | | | | | | | | | |  |
| --- | --- | --- | --- | --- | --- | --- | --- | --- | --- | --- | --- | --- | --- | --- |
| **Model** | |  | | **B** | **SE** | | **β** | | ***t*** | | ***p*** | | |  |
| H₀ |  | (Intercept) |  | 4.37 |  | 1.61 |  |  |  | 2.71 | |  | 0.01 |  |
| H₁ |  | (Intercept) |  | 5.88 |  | 8.09 |  |  |  | 0.73 | |  | 0.47 |  |
|  |  | Dosage |  | 1.13 |  | 1.45 |  | 0.1 |  | 0.78 | |  | 0.44 |  |
|  |  | Age |  | -0.004 |  | 0.42 |  | -0.001 |  | -0.01 | |  | 0.99 |  |
|  |  | Initial Vineland communication score |  | -0.08 |  | 0.07 |  | -0.14 |  | -1.15 | |  | 0.25 |  |

| **Supplementary table 8:**  Regression analysis predicting change in Vineland daily living skills scaled score using age, final dosage, and initial Vineland daily living skills scaled score of the participants as predictors. | | | | | | | | | | | | | |  |
| --- | --- | --- | --- | --- | --- | --- | --- | --- | --- | --- | --- | --- | --- | --- |
| **Model** | |  | | **B** | **SE** | | **β** | | ***t*** | | ***p*** | | |  |
| H₀ |  | (Intercept) |  | 4.01 |  | 1.47 |  |  |  | 2.72 | |  | 0.008 |  |
| H₁ |  | (Intercept) |  | 10.86 |  | 9.7 |  |  |  | 1.12 | |  | 0.27 |  |
|  |  | Dosage |  | -0.03 |  | 1.36 |  | -0.03 |  | -0.02 | |  | 0.98 |  |
|  |  | Age |  | -0.06 |  | 0.4 |  | -0.02 |  | -0.14 | |  | 0.89 |  |
|  |  | Initial Vineland daily living skills scaled score |  | -0.1 |  | 0.09 |  | -0.13 |  | -1.08 | |  | 0.28 |  |

| **Supplementary table 9:**  Regression analysis predicting change in Vineland socialization scaled score using age, final dosage, and initial Vineland socialization scaled score of the participants as predictors. | | | | | | | | | | | | | |  |
| --- | --- | --- | --- | --- | --- | --- | --- | --- | --- | --- | --- | --- | --- | --- |
| **Model** | |  | | **B** | **SE** | | **β** | | ***t*** | | ***p*** | | |  |
| H₀ |  | (Intercept) |  | 5.66 |  | 1.52 |  |  |  | 3.73 | |  | < .001 |  |
| H₁ |  | (Intercept) |  | 24.6 |  | 9.15 |  |  |  | 2.69 | |  | 0.009 |  |
|  |  | Dosage |  | 0.59 |  | 1.3 |  | 0.06 |  | 0.46 | |  | 0.65 |  |
|  |  | Age |  | -0.45 |  | 0.4 |  | -0.14 |  | -1.13 | |  | 0.26 |  |
|  |  | Initial Vineland socialization scaled score |  | -0.31 |  | 0.1 |  | -0.38 |  | -3.24 | |  | 0.002 |  |

| **Supplementary table 10:**  Regression analysis predicting change in Vineland total (ABC) scaled score using age, final dosage, and initial Vineland ABC scaled score of the participants as predictors. | | | | | | | | | | | | | |  |
| --- | --- | --- | --- | --- | --- | --- | --- | --- | --- | --- | --- | --- | --- | --- |
| **Model** | |  | | **B** | **SE** | | **β** | | ***t*** | | ***p*** | | |  |
| H₀ |  | (Intercept) |  | 4.37 |  | 1.18 |  |  |  | 3.7 | |  | < .001 |  |
| H₁ |  | (Intercept) |  | 5.52 |  | 7.67 |  |  |  | 0.72 | |  | 0.47 |  |
|  |  | Dosage |  | 0.87 |  | 1.09 |  | 0.1 |  | 0.8 | |  | 0.43 |  |
|  |  | Age |  | -0.03 |  | 0.32 |  | -0.01 |  | -0.1 | |  | 0.92 |  |
|  |  | Initial Vineland ABC scaled score |  | -0.05 |  | 0.08 |  | -0.08 |  | -0.7 | |  | 0.49 |  |

| **Supplementary table 11:**  Regression analysis predicting change in Cognitive blocks subtest score using age, final dosage, and initial Cognitive blocks subtest score of the participants as predictors. | | | | | | | | | | | | | |  |
| --- | --- | --- | --- | --- | --- | --- | --- | --- | --- | --- | --- | --- | --- | --- |
| **Model** | |  | | **B** | **SE** | | **β** | | ***t*** | | ***p*** | | |  |
| H₀ |  | (Intercept) |  | 0.13 |  | 0.25 |  |  |  | 0.52 | |  | 0.6 |  |
| H₁ |  | (Intercept) |  | 0.39 |  | 1.03 |  |  |  | 0.37 | |  | 0.71 |  |
|  |  | Dosage |  | 0.09 |  | 0.22 |  | 0.05 |  | 0.4 | |  | 0.69 |  |
|  |  | Age |  | -0.005 |  | 0.07 |  | -0.01 |  | -0.08 | |  | 0.94 |  |
|  |  | Initial Cognitive blocks subtest score |  | -0.08 |  | 0.05 |  | -0.18 |  | -1.55 | |  | 0.13 |  |

| **Supplementary table 12:**  Regression analysis predicting change in Cognitive matrix subtest score using age, final dosage, and initial Cognitive matrix subtest score of the participants as predictors. | | | | | | | | | | | | | |  |
| --- | --- | --- | --- | --- | --- | --- | --- | --- | --- | --- | --- | --- | --- | --- |
| **Model** | |  | | **B** | **SE** | | **β** | | ***t*** | | ***p*** | | |  |
| H₀ |  | (Intercept) |  | 0.14 |  | 0.19 |  |  |  | 0.76 | |  | 0.45 |  |
| H₁ |  | (Intercept) |  | 1.06 |  | 0.76 |  |  |  | 1.39 | |  | 0.17 |  |
|  |  | Dosage |  | -0.22 |  | 0.17 |  | -0.16 |  | -1.32 | |  | 0.19 |  |
|  |  | Age |  | 0.01 |  | 0.05 |  | 0.03 |  | 0.25 | |  | 0.8 |  |
|  |  | Initial Cognitive matrix subtest score |  | -0.11 |  | 0.05 |  | -0.28 |  | -2.39 | |  | 0.02 |  |

| **Supplementary table 13:**  Regression analysis predicting change in Cognitive vocabulary subtest score using age, final dosage, and initial Cognitive vocabulary subtest score of the participants as predictors. | | | | | | | | | | | | | |  |
| --- | --- | --- | --- | --- | --- | --- | --- | --- | --- | --- | --- | --- | --- | --- |
| **Model** | |  | | **B** | **SE** | | **β** | | ***t*** | | ***p*** | | |  |
| H₀ |  | (Intercept) |  | 0.29 |  | 0.28 |  |  |  | 1.03 | |  | 0.3 |  |
| H₁ |  | (Intercept) |  | 1.87 |  | 1.11 |  |  |  | 1.68 | |  | 0.1 |  |
|  |  | Dosage |  | -0.22 |  | 0.24 |  | -0.11 |  | -0.93 | |  | 0.36 |  |
|  |  | Age |  | -0.01 |  | 0.07 |  | -0.02 |  | -0.2 | |  | 0.84 |  |
|  |  | Initial Cognitive vocabulary subtest score |  | -0.18 |  | 0.06 |  | -0.32 |  | -2.86 | |  | 0.006 |  |

| **Supplementary table 14:**  Regression analysis predicting change in Cognitive verbal comprehension subtest score using age, final dosage, and initial Cognitive verbal comprehension subtest score of the participants as predictors. | | | | | | | | | | | | | |  |
| --- | --- | --- | --- | --- | --- | --- | --- | --- | --- | --- | --- | --- | --- | --- |
| **Model** | |  | | **B** | **SE** | | **β** | | ***t*** | | ***p*** | | |  |
| H₀ |  | (Intercept) |  | -0.04 |  | 0.22 |  |  |  | -0.18 | |  | 0.86 |  |
| H₁ |  | (Intercept) |  | -0.84 |  | 0.94 |  |  |  | -0.9 | |  | 0.37 |  |
|  |  | Dosage |  | 0.28 |  | 0.2 |  | 0.173 |  | 1.39 | |  | 0.17 |  |
|  |  | Age |  | 0.05 |  | 0.06 |  | 0.107 |  | 0.87 | |  | 0.38 |  |
|  |  | Initial Cognitive verbal comprehension subtest score |  | -0.1 |  | 0.06 |  | -0.186 |  | -1.6 | |  | 0.11 |  |

| **Supplementary table 15:**  Regression analysis predicting change in Cognitive coding subtest score using age, final dosage, and initial Cognitive coding subtest score of the participants as predictors. | | | | | | | | | | | | | |  |
| --- | --- | --- | --- | --- | --- | --- | --- | --- | --- | --- | --- | --- | --- | --- |
| **Model** | |  | | **B** | **SE** | | **β** | | ***t*** | | ***p*** | | |  |
| H₀ |  | (Intercept) |  | 0.22 |  | 0.18 |  |  |  | 1.26 | |  | 0.21 |  |
| H₁ |  | (Intercept) |  | 0.65 |  | 0.72 |  |  |  | 0.91 | |  | 0.37 |  |
|  |  | Dosage |  | -0.02 |  | 0.15 |  | -0.01 |  | -0.1 | |  | 0.92 |  |
|  |  | Age |  | 0.003 |  | 0.04 |  | 0.01 |  | 0.06 | |  | 0.95 |  |
|  |  | Initial Cognitive coding subtest score |  | -0.14 |  | 0.05 |  | -0.29 |  | -2.59 | |  | 0.01 |  |
